# Redefining digital health interfaces with large language models

**DOI:** 10.3389/frai.2025.1623339

**Published:** 2025-09-26

**Authors:** Fergus Imrie, Paulius Rauba, Mihaela van der Schaar

**Affiliations:** ^1^Department of Statistics, University of Oxford, Oxford, United Kingdom; ^2^Department of Applied Mathematics and Theoretical Physics, University of Cambridge, Cambridge, United Kingdom

**Keywords:** large language model (LLM), risk score, cardiovascular disease, LLM agents, digital health

## Abstract

Digital health tools have the potential to significantly improve the delivery of healthcare services. However, their adoption remains comparatively limited due, in part, to challenges surrounding usability and trust. Large Language Models (LLMs) have emerged as general-purpose models with the ability to process complex information and produce human-quality text, presenting a wealth of potential applications in healthcare. Directly applying LLMs in clinical settings is not straightforward, however, as LLMs are susceptible to providing inconsistent or nonsensical answers. We demonstrate how LLM-based systems, with LLMs acting as agents, can utilize external tools and provide a novel interface between clinicians and digital technologies. This enhances the utility and practical impact of digital healthcare tools and AI models while addressing current issues with using LLMs in clinical settings, such as hallucinations. We illustrate LLM-based interfaces with examples of cardiovascular disease and stroke risk prediction, quantitatively assessing their performance and highlighting the benefit compared to traditional interfaces for digital tools.

## 1 Introduction

Digital healthcare technologies represent a frontier in medicine. Despite a multitude of tools being developed (Sutton et al., 2020; Dunn et al., 2018), clinical adoption of such methods faces significant hurdles (Eichler et al., 2007; Mathews et al., 2019), with some even calling their use “infeasible” (Müller-Riemenschneider et al., 2010) and “substantially conceptual” (Abernethy et al., 2022). A key issue is usability (Ratwani et al., 2019), which can result in errors associated with patient harm (Howe et al., 2018) and contribute to clinician frustration, jeopardizing patient safety (Shanafelt et al., 2016; Gardner et al., 2019). New tools employing artificial intelligence (AI) and machine learning offer substantial promise, with their impact expected to be felt across all areas of healthcare (Bajwa et al., 2021). However, these approaches face the same usability challenges as existing digital tools, while introducing additional questions about model trust (Rajpurkar et al., 2022; Asan et al., 2020). Consequently, these issues have limited the uptake and impact of AI technologies in clinical settings thus far (Goldfarb and Teodoridis, 2022; Davenport and Kalakota, 2019; Kelly et al., 2019).

Several approaches have sought to simplify or automate the process of obtaining predictions from clinical predictive models to improve their usability. These include points-based scoring systems (Gage et al., 2001), web-based calculators (Hippisley-Cox et al., 2017; Imrie et al., 2023a), and integration within electronic health records (Rothman et al., 2013). While this can make such tools easier to use, simply obtaining a prediction is frequently insufficient and more is required to build model trust with clinicians (Rajpurkar et al., 2022) and regulators (Food and Drug Administration and others, 2019; Mourby et al., 2021). For example, dynamic interactions in the form of natural language dialogues that can adapt to the specific needs of individual clinicians and patients have been identified as a key feature for effectively deploying machine learning models in healthcare (Lakkaraju et al., 2022).

Large Language Models (LLMs) offer a potential solution to the challenges faced by digital tools. LLMs have recently captured the imagination of both the research community and the general public, pushing the boundaries of human-machine interaction. Consequently, there is great interest in applying LLMs in healthcare, with potential applications including facilitating clinical documentation, summarizing research papers, or as a chatbot for patients (Moor et al., 2023).

Applying LLMs in safety-critical clinical settings is not straightforward. LLMs may provide inconsistent or nonsensical answers (Singhal et al., 2023; Lecler et al., 2023) and have a tendency to hallucinate facts (Maynez et al., 2020; Ji et al., 2023), which is unacceptable when making high-stakes clinical decisions. Additionally, LLMs can encounter difficulty with seemingly basic functionality, such as mathematical calculations or factual lookup (Patel et al., 2021; Schick et al., 2023), and are unable to access up-to-date information by default (Komeili et al., 2022). These limitations constrain the utility of directly applying LLMs in medicine.

**Figure 1:**
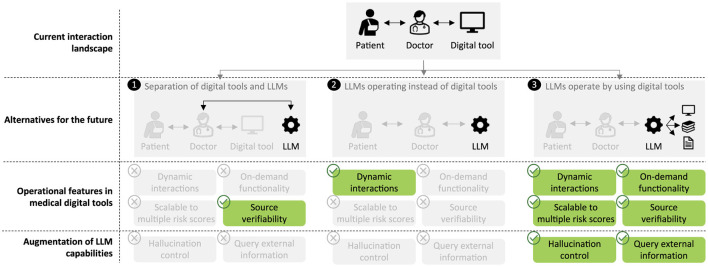
Clinicians have previously needed to interact directly with digital tools, such as risk scores. While others have discussed LLMs replacing existing clinical tools (1, 2), we envisage LLMs forming a novel interface by enabling dynamic interactions and facilitating deeper engagement with tools and related information, such as explainability, medical papers, and clinical guidelines (3).

In this paper, we explore a new application of LLMs in healthcare by proposing their use as facilitators of clinician interactions with AI models and digital tools. We construct LLM-based systems that provide intuitive natural language interfaces. These systems enable dynamic, adaptable dialogues that cater to the specific needs of clinicians and patients. This addresses limitations of existing pre-specified interfaces in healthcare and conceptually differs from previous applications of LLMs in healthcare, such as training medicine-specific LLMs (Luo et al., 2022; Yang et al., 2022) or using LLMs for prediction (Li et al., 2020; Steinberg et al., 2021; Jiang et al., 2023). The ability of our LLM-based approach to adapt and tailor interactions represents a significant advance in the functionality of such tools, improving efficiency and usability (Figure 1).

We first describe our approach. LLMs do not inherently possess the ability to access external tools or information. We propose augmenting the base functionality of LLMs to enable them to access approved medical tools and other sources of information, thereby not solely relying on the inherent capabilities of a given LLM and using the LLM as an agent. Our framework is scalable to multiple predictive models, unifying digital tools within a single, natural language-based interface. By adopting a systems approach, the LLM does not itself issue predictions and can access relevant domain-specific information, rather than needing to possess specific knowledge. Consequently, the potential for hallucinations is reduced and we ensure actionable information is provided by approved clinical sources.

To demonstrate our approach, we examine risk scoring and primarily consider cardiovascular disease (CVD), the most common cause of mortality globally (Muthiah et al., 2022). Primary prevention programs use prognostic models, such as the Framingham score (D'Agostino et al., 2008) in the United States, SCORE2 (SCORE2 working group and ESC Cardiovascular risk collaboration, 2021) in Europe, and QRisk3 (Hippisley-Cox et al., 2017) in the UK, to estimate the risk of developing CVD. This allows high-risk individuals to be identified and their risk to be managed via interventions, such as lifestyle modifications or pharmaceuticals. We construct two LLM-based systems for interacting with CVD risk scores and accessing related information based on a traditional risk score and a machine learning model. We additionally construct a LLM-based system for interacting with the CHA_2_DS_2_-VASc score (Lip et al., 2010) that assesses stroke risk in patients with atrial fibrillation. We then quantitatively validate the effectiveness of our LLM-based approach on two diverse sets of questions, each comprising over 100 questions that cover 11 representative scenarios across various stages of risk estimation. Finally, we provide several examples of dynamic interactions that substantially extend the capabilities of existing fixed interfaces to illustrate the potential impact of our proposed approach.

## 2 Methods

### 2.1 LLM-based interfaces

**Figure 2:**
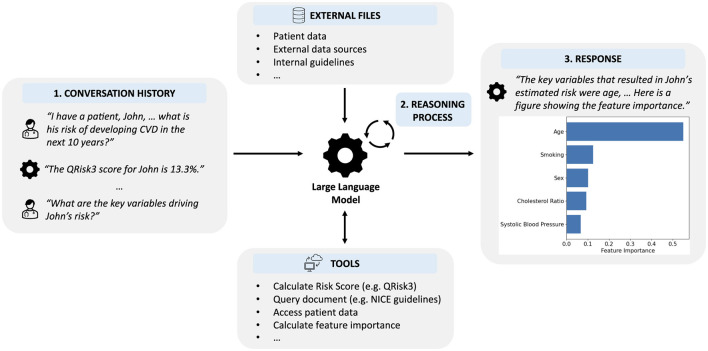
Overview of our LLM-based system that enables clinicians to interface with digital tools using natural language inputs. (1) The LLM is provided with the history of the interaction, including the current request. (2) Using an iterative reasoning process, the LLM decides which, if any, tools are required and with what input. (3) The LLM provides a response to the user incorporating information provided by any tools that were used.

In this section, we describe our LLM-based system incorporating digital health tools. While LLMs are powerful models for natural language processing, they inherently lack the functionality to utilize external tools or access additional information. Methods to extend LLMs beyond text generation are in their infancy but can already be used to significantly expand the capabilities of LLMs (Schick et al., 2023; Nakano et al., 2021). Instead of using an LLM to issue predictions or provide information directly, we developed an LLM-based system unifying numerous external tools, sources of information, and clinical data within a single, natural language-based interface (Figure 2).

#### 2.1.1 LLM framework

By default, LLMs provide responses in the form of text based on the provided context, such as a prompt or conversation history. To construct interfaces for digital tools using LLMs, we instead viewed the LLM as an agent that can interact with an environment to solve tasks. Formally, at each step *t*∈*T*, the agent receives observation *o*_*t*_∈*O* from the environment and subsequently takes action *a*_*t*_∈*A* according to policy π(*a*_*t*_|*h*_*t*_), where *h*_*t*_ = (*o*_0_, *a*_0_, …, *o*_*t*−1_, *a*_*t*−1_, *o*_*t*_) is the history. To enable the agent to both reason and use external tools, we used the ReAct framework (Yao et al., 2023) which decomposes the action space as Â = *A*∪*L*, where *a*∈*A* are actions using specific tools and an action *a*∈*L* represents not using an external tool but instead allows the model to reason over the history about what action to take next.

Since we will provide the agents with tasks in the form of natural language, and actions in the language space *L* are (essentially) infinite, we chose to benefit from strong language priors and use a pretrained LLM. To demonstrate the versatility of our approach, we implemented our LLM-based interfaces using off-the-shelf pretrained LLMs, specifically GPT-4 (OpenAI, 2023) and GPT-4o (OpenAI, 2024). Interactions with external tools were implemented using LangChain (Chase, 2022).

Frameworks such as Toolformer (Schick et al., 2023) and WebGPT (Nakano et al., 2021) trained LLMs to use basic tools, such as calculators, calendars, and search engines, via self-supervised fine-tuning and fine-tuning using behavior cloning and reinforcement learning, respectively. In contrast, following ReAct (Yao et al., 2023), we employed in-context learning (Dong et al., 2024) in the form of prompting, providing the LLM with sufficient information about possible actions and using the underlying reasoning capabilities of the LLM. For each tool that the LLM is able to use, a short description of the tool, application scenarios, and the input and output formats are provided as text. We did not perform any prompt optimization. Using in-context learning removes the need for further training of the LLM, which might be challenging in the medical domain without suitable examples, and readily enables multiple tools to be used and new tools to be added, unlike other frameworks (Schick et al., 2023), ensuring the approach is flexible and extendable.

Interactions with the LLM-based system are via a simple text-based entry and responses can be both text and images, depending on the tool used, with user interfaces built using StreamLit (Streamlit, n.d). Additionally, we implemented a “source” functionality that allows the user to see whether the LLM used a tool or accessed specific information and, if so, which tool and with what input. This helps avoid hallucinations since it enables verification that the information was issued by an underlying clinical tool or source rather than being generated by the LLM.

#### 2.1.2 External tools

We constructed two illustrative LLM-based systems for cardiovascular disease risk scores: one for an existing clinical risk score, QRisk3 (Hippisley-Cox et al., 2017), and another for a machine learning-based risk score. We also constructed an LLM-based system for interacting with the CHA_2_DS_2_-VASc score (Lip et al., 2010) that assesses stroke risk in patients with atrial fibrillation. Below, we describe the external tools and sources of information made available to the LLM in each case.

##### 2.1.2.1 QRisk3 interface

In our first instantiation of an LLM-based system, we show how LLMs can incorporate existing tools and information for CVD risk prediction. We provided the LLM access to QRisk3 (Hippisley-Cox et al., 2017), a risk prediction tool that assesses the likelihood of developing CVD within 10 years. We enabled the LLM to use the risk score either with the provided data or, if requested by the user, to modify a variable and assess the impact of such a change on the patient's risk. Additionally, we provided the LLM with access to the academic paper describing QRisk3 (Hippisley-Cox et al., 2017) and the National Institute for Health and Care Excellence (NICE) clinical guidelines for CVD (National Institute for Health and Care Excellence, 2014).

##### 2.1.2.2 AutoPrognosis interface

In our second example of an LLM-based system, we equipped the LLM with a CVD risk prediction model developed using AutoPrognosis 2.0 (Imrie et al., 2023a). To help build model trust, a critical step for clinical prognosis models (Rajpurkar et al., 2022; Asan et al., 2020), we enabled the LLM to use explainable AI (XAI) methods (Imrie et al., 2023b) on the underlying model, allowing users to investigate the rationale for predictions, both in general and for the specific patient. In particular, we used SHAP (Lundberg and Lee, 2017) to interpret model predictions. We additionally provided the LLM with a document containing information about the variables used by the risk score, the underlying methodology and how the model was constructed, and details regarding the cohort used to develop the model.

##### 2.1.2.3 CHA_2_DS_2_-VASc interface

Finally, we constructed an LLM-based system for a different risk prediction problem: stroke risk in patients with atrial fibrillation. We provided the LLM access to CHA_2_DS_2_-VASc (Lip et al., 2010), a rules-based score that assesses the annual risk of stroke and thromboembolism in patients with atrial fibrillation. As above, we enabled the LLM to use the risk score either with the provided data or, if requested by the user, to modify a variable and assess the impact on the score. Additionally, we provided the LLM with the academic paper describing CHA_2_DS_2_-VASc (Lip et al., 2010) and the NICE clinical guidelines for atrial fibrillation (National Institute for Health and Care Excellence, 2021).

### 2.2 Quantitative assessment of LLM-based systems

Clinicians and medical practitioners are faced with a multitude of questions when using risk scores beyond simply obtaining the risk for a given patient. Currently, existing interfaces primarily (and often only) enable the patient's risk to be calculated. To quantify the extent to which LLM-based interfaces with access to external information and tools could benefit risk estimation, we detailed 11 representative questions covering four distinct stages of a patient encounter (Table 1).

**Table 1:** Representative questions that a clinician might have relating to a risk score at different stages of a patient encounter, together with whether existing interfaces for risk scores provide this information.

**Stage**	**Representative questions**	**Existinginterfaces**
Before	Which features does the risk score use?	×
Patient	Why are these features included in the risk score?	×
Encounter	How was the risk score validated?	×
	What is the methodology underlying the risk score?	×
Before risk	When do clinical guidelines recommend risk scoring?	×
Scoring	What is the recommended risk score?	×
	Who is the risk score suitable for?	×
During risk	What is the risk for this patient?	✓
Scoring	What characteristics led to the patient's risk?	×
	What effect would changing this feature have on the risk?	×
After risk scoring	What action is recommended by the guidelines based on the risk?	×

All questions can be addressed using LLM-based interfaces.

The first stage is before any patient encounter to enable healthcare practitioners to better understand a particular risk score. This includes understanding which clinical variables are used, how the risk score was developed, and for what patient population the risk score is applicable. This is also an area where the inherent knowledge LLMs possess can prove beneficial beyond simply facilitating information retrieval; for example, if the user was not familiar with a particular modeling approach, they could ask the LLM for more details. Second, before conducting risk scoring, clinicians must understand when and for whom the guidelines recommend risk scoring, as well as which score to use. Third, beyond just receiving the output from a risk score, the clinician or patient might want to better understand the rationale for the prediction or the impact of specific features on the predicted risk. Lastly, after receiving a risk estimate, the healthcare professional and patient need to know what possible actions are recommended by the guidelines given the output of the risk score.

To quantitatively assess the performance of LLM-based systems and demonstrate their suitability to be deployed in such scenarios, we created a set of 127 specific questions across the 11 representative questions for CVD and 106 questions for the atrial fibrillation scenario. Unless otherwise stated, each question was asked once to each LLM or LLM-based system. Responses were assessed according to a specific set of criteria for each question and were also checked for hallucinations. Responses that correctly answered the question with no hallucinations were deemed successful.

A key component of the proposed LLM-based system is the ability to access external information and tools. Thus, in addition to assessing our LLM-based system, we posed the same questions to a standalone LLM. We chose to compare to the same LLMs that we used to implement our LLM-based systems, and thus any differences are being driven specifically by the ability to query and interact with external tools and sources of information. For a full list of all questions and responses for each system considered, see Code and Data Availability.

## 3 Results

In this section, we first quantitatively evaluate our LLM-based systems and then provide multiple examples that demonstrate how such systems can provide a novel interface for digital health tools, in particular clinical risk scores.

### 3.1 Performance of LLM-based interfaces

We compared our LLM-based systems, which enable the use of tools and can access external information, with LLMs without such capabilities. Our LLM-based systems that use external tools and sources of information each successfully answered all but one question across 127 questions encompassing 11 representative situations in CVD risk prediction, achieving an overall success rate of over 99% (Table 2, Supplementary Table S1), and correctly answered 104 of 106 questions in stroke risk prediction in atrial fibrillation patients (98%, Supplementary Table S2). In comparison, the standalone LLMs answered around half the questions correctly in the CVD risk prediction scenario (GPT-4: 44%, GPT-4o: 50%) and 79 of 106 questions correctly in the atrial fibrillation scenario (75%). This demonstrates the importance of external functionality and information beyond the base LLM.

**Table 2:** Performance of LLM-based interfaces using GPT-4 for CVD risk prediction.

	**Representative questions**	**GPT-4**	**Ours**
Q1	Which features does the risk score use?	0/10 (0.0%)	10/10 (100%)
Q2	Why are these features included in the risk score?	20/21 (95.2%)	21/21 (100%)
Q3	How was the risk score validated?	5/10 (50.0%)	9/10 (90.0%)
Q4	What is the methodology underlying the risk score?	5/10 (50.0%)	10/10 (100%)
Q5	When do clinical guidelines recommend risk scoring?	0/10 (0.0%)	10/10 (100%)
Q6	What is the recommended risk score?	10/10 (100%)	10/10 (100%)
Q7	Who is the risk score suitable for?	6/10 (60.0%)	10/10 (100%)
Q8	What is the risk for this patient?	0/10 (0.0%)	10/10 (100%)
Q9	What characteristics led to the patient's risk?	0/10 (0.0%)	10/10 (100%)
Q10	What effect would changing this feature have on the risk?	0/10 (0.0%)	10/10 (100%)
Q11	What is recommended by the guidelines based on the risk?	10/16 (62.5%)	16/16 (100%)
	**Overall**	56/127 (44.1%)	126/127 (99.2%)

Across a varied set of questions, our LLM-based system (Ours) significantly outperformed the same LLM used in a standalone manner (GPT-4). For each representative question, we report the number of successes and questions, as well as the success rate.

**Figure 3:**
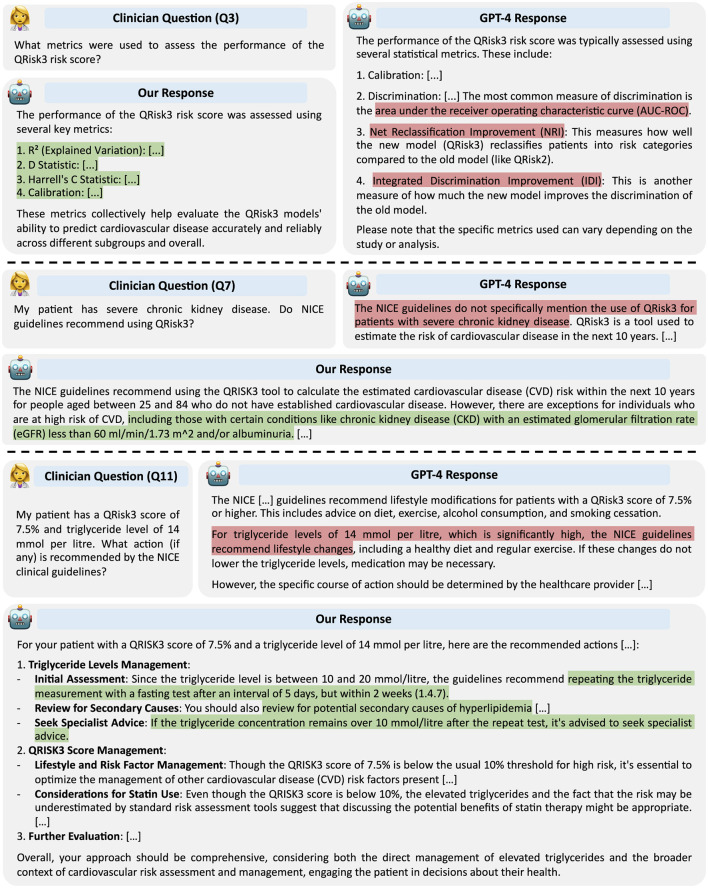
Example responses from GPT-4 and our LLM-based system. Responses highlighted in red contain inaccuracies; those in green are correct.

In the remainder of this section, we focus our analysis on the LLM-based system for CVD risk prediction using GPT-4 as the underlying LLM. However, similar trends were observed in each scenario. Our LLM-based system was able to cope equally well with all types of questions (Table 2), while there was significant variability in the performance of the standalone GPT-4 model, which performed well on questions that required more straightforward or general answers (e.g. Q2, Q6), significantly less well on questions that required more detailed or nuanced answers (e.g. Q1, Q3, Q4, Q11), and could not answer questions that require external tools (i.e. Q8, Q9, Q10). Three example responses for each approach illustrating some of these differences are provided in Figure 3.

Most failures of the standalone LLM that were not a direct consequence of an inability to use tools fell into one of the following classes: (1) hallucinating specific factual information, (2) an inability to access up-to-date information due to a fixed knowledge cutoff, and (3) a lack of deeper understanding in nuanced situations.

Hallucinations are a significant barrier to the successful adoption of LLMs in medicine, degrading performance and creating substantial safety concerns (Ji et al., 2023). Our findings suggest that enabling LLMs to access external data reduces the risk of hallucination. In our experiments, we did not find evidence of our LLM-based system hallucinating in any response, while the standalone LLM suffered from frequent hallucinations across multiple questions.

This is exemplified by Question 1, which requires the LLMs to provide a list of the 21 features used in QRisk3. In all responses, our LLM-based system with access to the QRisk3 publication correctly provided the exact features used. In contrast, while GPT-4 typically provided around 20 of the correct features, in all instances at least one feature was omitted and in nine of ten cases features were hallucinated. These errors ranged from more subtle, for example including HIV or AIDS status, which was considered as input for QRisk3 but ultimately not included (Hippisley-Cox et al. 2017), to features not mentioned in the QRisk3 paper, such as polycystic ovary syndrome, postpartum psychosis, and asthma.

Similarly, in responses to Questions 3 and 4, GPT-4 often failed to provide specific details or hallucinated, while our LLM-based system was able to extract the relevant details from the provided information. As an example, when asked which metrics were used to assess QRisk3, our LLM-based system correctly provided the four metrics used, while GPT-4 did not specify how calibration was measured, suggested an incorrect discrimination metric, and erroneously claimed two other metrics were calculated (Figure 3).

The second significant limitation to using fixed LLMs without access to external knowledge is an inherent knowledge cutoff. This is most clearly demonstrated by Question 5, which asked when the NICE Clinical Guidelines (National Institute for Health and Care Excellence, 2014) recommend risk scoring. Before May 2023, the NICE Clinical Guidelines specified that “People older than 40 should have their estimate of CVD risk reviewed on an ongoing basis” and “Use the QRISK2 risk assessment tool to assess CVD risk for the primary prevention of CVD in people up to and including age 84 years.” In May 2023, the NICE Clinical Guidelines were updated, specifying to “Use the QRisk3 tool to calculate the estimated CVD risk within the next 10 years for people aged between 25 and 84 without CVD” and “Review estimates of CVD risk on an ongoing basis for people over 40.”

GPT-4, which has a knowledge cutoff of April 2023, consistently responded that the guidelines recommended risk scoring only in adults aged 40–84. Our LLM-based system, despite employing GPT-4 as the underlying LLM, provided the correct age range for QRisk3. To check that this was a consequence of the training data, we additionally tested an LLM trained on more recent data. Specifically, we used GPT-4o, which has a knowledge cutoff of October 2023 and thus has been trained on data after the updated guidelines. GPT-4o correctly states the eligibility range for QRisk3; however, the limitation of a fixed knowledge cutoff is clear.

The third significant failure mode of the standalone LLM was exhibited when the questions required more specific details or nuance. For Questions 7 and 11, GPT-4 achieved around 60% success primarily by correctly answering the questions involving the most straightforward and general criteria. However, when more specific information was required, the standalone GPT-4 model was not able to correctly answer. For example, the NICE guidelines (1.4.7) specify a specific set of actions for individuals with elevated triglyceride levels (10–20 mmol/l). GPT-4 correctly noted the patient's triglyceride level of 14 mmol/l was high but did not provide the specific actions to take (Figure 3). In contrast, our LLM-based system, which could query the guidelines, successfully answered such questions.

Beyond these failure modes, GPT-4 declined to answer in three cases and said it could only provide general advice twice. Further, in nine of the ten responses to Question 10, GPT-4 stated that the exact impact depended on the specific model, also not providing a specific answer in the other case. While this can be seen as better than confidently providing an incorrect answer, it still represents an inability to answer the question successfully, unlike the augmented LLM-based system, which answered all questions correctly.

The only question in our assessment that the LLM-based system answered incorrectly was “Which data sources were employed to create and verify the QRisk3 risk score?” (part of Question 3), where it did not explicitly state that the QResearch database was used, instead responding “The QRisk3 risk score was created and verified using data from general practice records, mortality records, and hospital admission records. These data sources are linked, providing a comprehensive view of patient health outcomes, which helps in accurately determining the incidence of cardiovascular disease among the study cohorts.” Since LLM sampling can be performed in a stochastic manner, we regenerated the response to this question, which resulted in a correct answer.

The errors of the LLM-based systems using GPT-4o as base models were similar. For example, the two errors in the atrial fibrillation scenario were not specifying precise thresholds for low, medium, and high risk designations and, in one case, not providing all possible scenarios in which the CHA_2_DS_2_-VASc risk score should be used. In contrast to the CVD risk prediction scenario, the standalone LLM exhibited improved performance on the questions for stroke risk prediction in patients with atrial fibrillation. This was primarily a result of CHA_2_DS_2_-VASc being a relatively simple points-based score, which the base LLM had learnt. The standalone LLM was frequently able to successfully apply the CHA_2_DS_2_-VASc criteria to calculate risk (Q8 and Q10) and determine the most important risk factors (Q9). However, it did not always perform calculations correctly and exhibited similar failure modes on the other questions as in the CVD risk scoring scenario.

Overall, our quantitative assessment of standalone LLMs and our LLM-based system clearly demonstrates the benefits of augmenting LLMs with additional functionality and information, in particular for reducing hallucinations.

### 3.2 Illustrative use cases of LLM-based interfaces

Having quantitatively assessed the ability of an LLM-based system to answer a diverse range of questions related to clinical risk scoring, we now provide several multi-stage examples of how such systems could be used in practice to provide a novel interface for digital health tools, specifically clinical risk scores.

#### 3.2.1 QRisk3 interface

**Figure 4:**
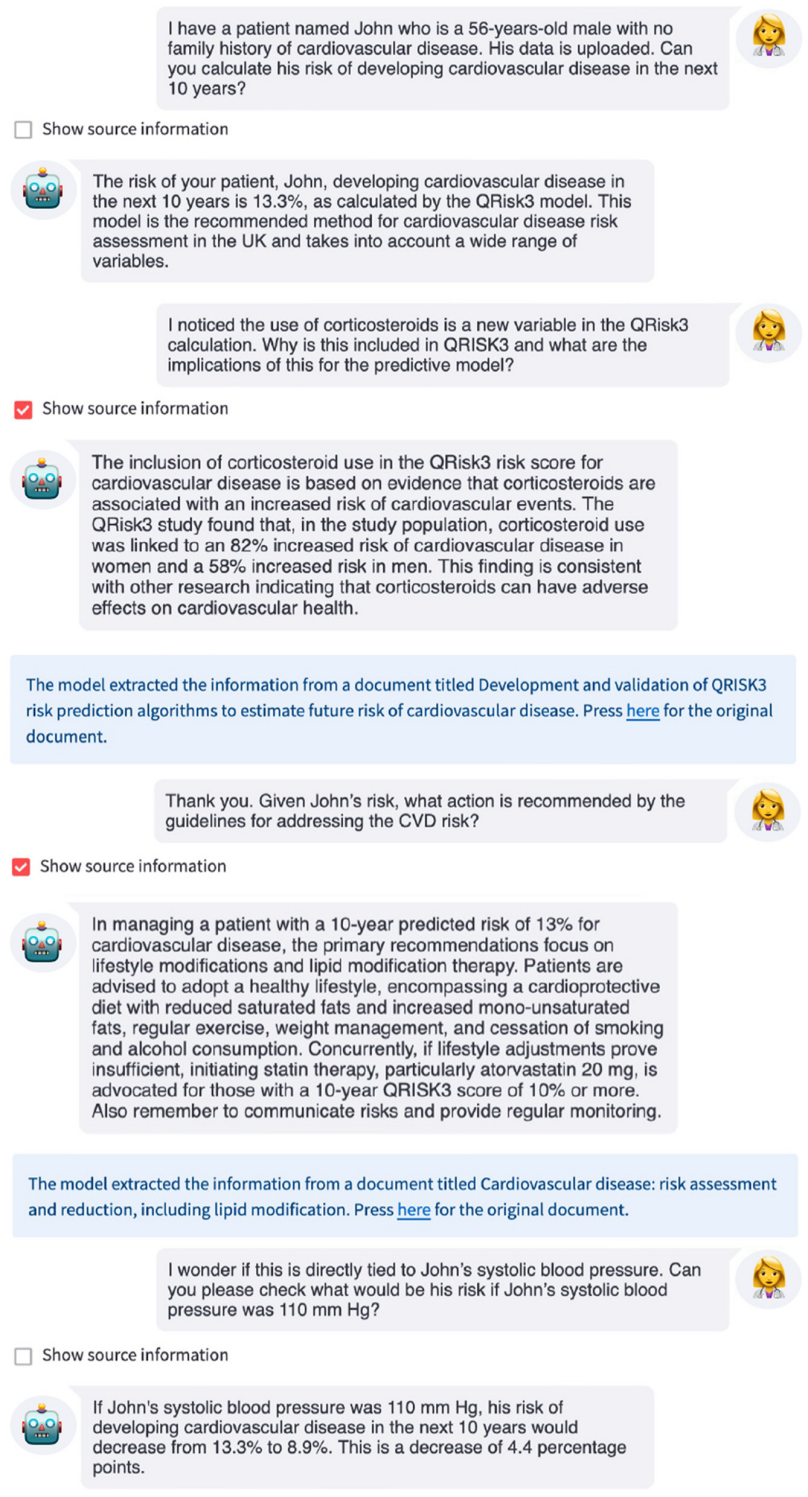
Example interaction with an LLM-based interface for QRisk3. The LLM uses QRisk3 to calculate the patient's 10-year risk of developing CVD, explains why certain features are included in the risk score using the QRisk3 paper (Hippisley-Cox et al., 2017), provides recommendations from the NICE clinical guidelines, and recalculates the patient's risk under a counterfactual scenario.

We first provide an illustrative conversation with the LLM-based system that has access to the QRisk3 model, the academic paper describing QRisk3 (Hippisley-Cox et al., 2017), and the National Institute for Health and Care Excellence (NICE) clinical guidelines for CVD (National Institute for Health and Care Excellence, 2014; Figure 4).

In this example, when asked for the patient's 10-year risk of developing CVD, the LLM used QRisk3 to estimate the patient's risk, providing this to the user. The LLM then summarized the relevant information from the QRisk3 paper to explain the inclusion of certain features before providing the recommended action for this patient based on the NICE clinical guidelines. Finally, the LLM used QRisk3 to recalculate the patient's counterfactual risk assuming that they were able to reduce their systolic blood pressure to within normal ranges. This allows both the clinician and patient to understand the potential impact of changes to modifiable variables on the patient's risk. While the clinician could have used the underlying resources to obtain this information, the LLM interface made the interaction simpler and more efficient, which has been identified as a key limitation of digital tools (Ratwani et al., 2019; Mathews et al., 2019). Furthermore, studies have shown that even with access to the relevant documents, time limitations often prevent clinicians finding the correct answers or even pursuing answers to their questions, which can lead to suboptimal patient care decisions (Del Fiol et al., 2014; Kell et al., 2024), while readily-available, accurate, up-to-date information could improve adherence to clinical guidelines (Cabana et al., 1999; Ament et al., 2015).

#### 3.2.2 AutoPrognosis interface

**Figure 5:**
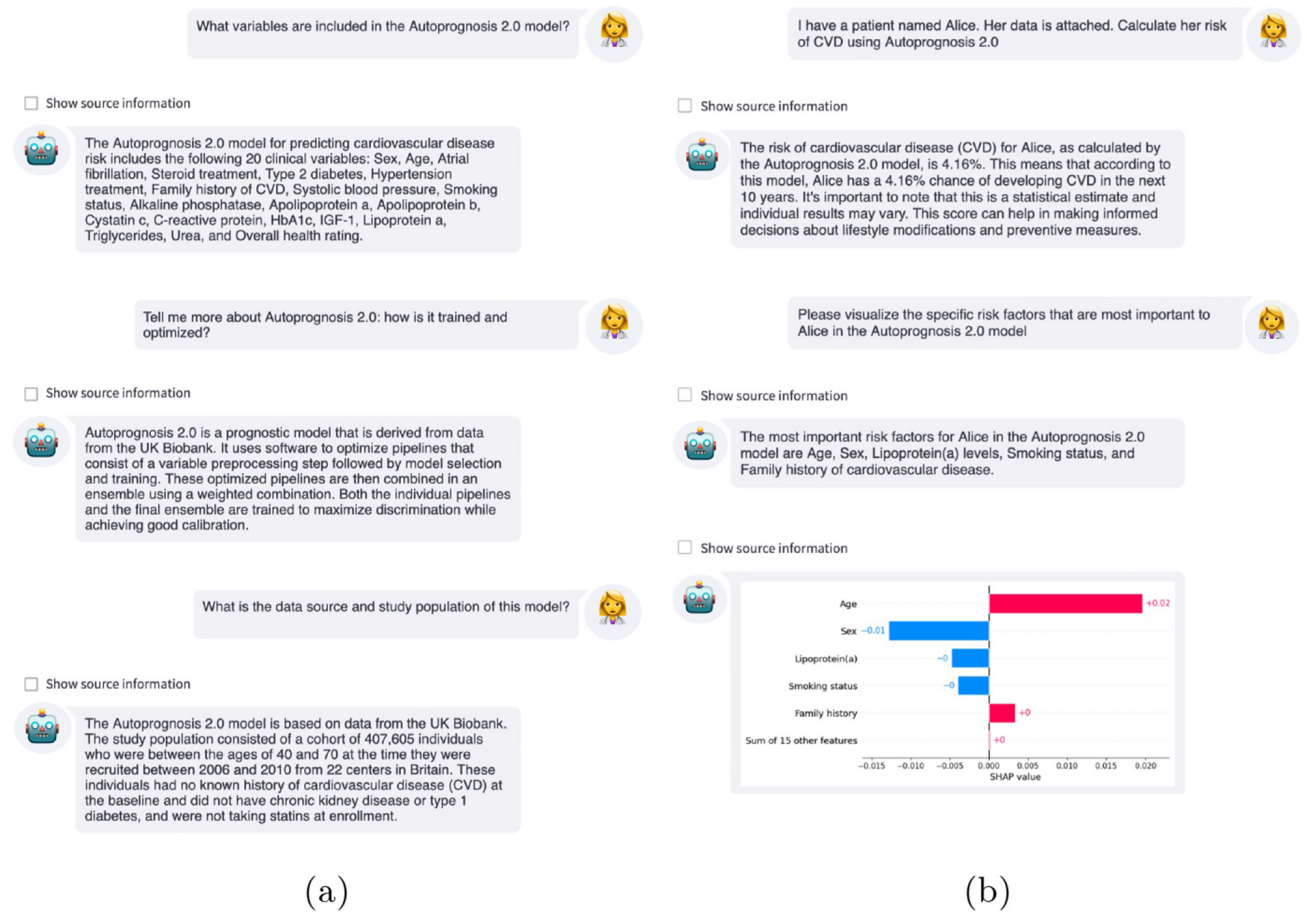
Example interactions with LLM-based interfaces. **(a)** Clinicians can ask the LLM questions about the AutoPrognosis risk score, such as what variables are included, the underlying methodology, and the cohort on which the model was derived. **(b)** After calculating the patient's risk using the AutoPrognosis model, the clinician can query why this prediction was issued using explainable AI to improve understanding of the model predictions.

We additionally present two example interactions using the LLM-based interface with access to the machine learning CVD risk score developed using AutoPrognosis (Figure 5). In our first example, we demonstrate the role such an interface could have upon model deployment (Figure 5a). A clinician might have many questions about a risk score before using the predictions to inform recommendations or interventions (Table 1). Here, the LLM provided the user with specific information about the variables used by the risk score, the underlying methodology, how the model was constructed, and the cohort of individuals used to train the model. This can enable a user to rapidly learn about a new model, facilitating successful deployment.

Building model trust is a crucial step for prognostic models, in particular for models that are not inherently interpretable (Rajpurkar et al., 2022). A recent study found that medical decision-makers had a strong preference for interactive explanations and, in particular, for these interactions to take the form of natural language dialogues (Lakkaraju et al., 2022).

After calculating the patient's risk using the AutoPrognosis model, the LLM-based system used Shapley additive explanations (SHAP) (Lundberg and Lee, 2017) to help the clinician understand why the model issued this prediction. As shown in Figure 5b, the estimated 4.2% risk for this individual was primarily caused by their age and family history of CVD, mitigated by being a woman, not smoking, and low levels of lipoprotein (a). An additional interaction can be found in Supplementary Figure S2.

Finally, due to large-scale pretraining, LLMs possess general knowledge of many topics; this can provide valuable additional information during interactions beyond the specific tools and external information sources provided to the LLM. For example, if a clinician is not familiar with the underlying XAI methodology, SHAP (Lundberg and Lee, 2017), the LLM could explain how the approach works in a variety of different ways and possibly over multiple interactions with the clinician, allowing specific queries or misunderstandings to be clarified. The underlying knowledge of LLMs extends the utility of LLM-based interfaces beyond simply using existing tools.

## 4 Discussion

Large Language Models hold substantial promise for the medical domain, particularly in augmenting digital workflows and improving the efficiency and effectiveness of healthcare delivery. The ability to integrate external tools and functionality with LLMs paves the way for innovative applications and can overcome limitations of LLMs, such as hallucinations (Ji et al., 2023). Doing so offers a potential transformation for how clinicians interact with digital tools and sources of information, helping overcome the challenges of deploying clinical AI models.

We have demonstrated how LLMs can provide a unique interface between healthcare professionals and clinical predictive models, such as risk scores, by acting as agents within an LLM-based system. Currently, clinicians must access these tools via fixed user interfaces or application programming interfaces (APIs), with existing interfaces typically only calculating risk. We have showed how, through an LLM-based interface, practitioners can obtain substantial additional information about the risk score, its development and methodology, the prediction issued, and related medical guidelines in a manner that specifically addresses their needs or questions without providing superfluous information.

In particular, we developed LLM-based interfaces for QRisk3 (Hippisley-Cox et al., 2017), the current recommended risk score in the UK for CVD, a machine learning-based risk score for CVD, and CHA_2_DS_2_-VASc (Lip et al., 2010), which is recommended for risk assessment for stroke in patients with atrial fibrillation. We quantitatively assessed the performance of our LLM-based systems and compared its capabilities with standalone LLMs. We also provided several illustrative examples of more complex multi-step use cases, demonstrating the potential of such approaches at various stages of a patient encounter. Our approach is scalable and does not require any additional training of the language model, although approaches that improve with use could be yet more powerful. Additionally, we aim to mitigate the problem of hallucination by ensuring that actionable advice is anchored in approved clinical resources, contrasting several previous applications of LLMs in medicine that focused exclusively on the knowledge and information learned by LLMs. While our approach does not guarantee that hallucinations cannot occur, our empirical analysis found substantially fewer examples of hallucinations for LLM-based systems than standalone LLMs.

In this paper, we have focused on clinicians interacting with digital tools. However, there are numerous stakeholders in healthcare in addition to clinicians, such as patients, regulators, and administrators, each with different goals and requirements (Imrie et al., 2023b). For example, in concurrent work, Shi et al. developed a retrieval-augmented generation instantiation of ChatGPT to help patients with adolescent idiopathic scoliosis and their families prepare for discussions with clinicians (Shi et al., 2023). Our framework and approach could be applied to improve digital health interfaces for these alternate stakeholders. While this could have additional challenges, there are potentially even more substantial benefits for such individuals, given the differences in requirements, knowledge, and familiarity with digital health technology, among other factors.

Despite the general capabilities of LLMs, they can lack domain-specific knowledge. This has led to the development of medical-focused LLMs, either by training new LLMs from scratch (Luo et al., 2022; Taylor et al., 2022; Yang et al., 2022) or by adapting existing general-purpose LLMs (Singhal et al., 2023). While we showed using specialist LLMs is not required, they could be readily incorporated due to the modularity of our approach, possibly further enhancing the functionality of LLM-based interfaces. Additionally, although we demonstrated that in-context learning is effective, fine-tuning LLMs for specific interfaces could further improve their task-specific capabilities, albeit this would add complexity to the creation of LLM-based interfaces. Finally, we expect the continued advances in LLMs, such as improved base models or ways of accessing external tools and information, will complement the use case of LLMs described in our work and should make them more performant at such tasks.

While our experiments highlight the promise of LLM-based interfaces, several additional considerations must be taken into account before deploying such systems. From a technical perspective, LLMs require more computational resources than previous interfaces to risk scores. The cost of LLM-based systems is rapidly declining; at current OpenAI API pricing, we estimate an interaction would cost less than $0.10, while local systems could prove even more affordable. We believe these expenses could be more than offset by productivity gains. Additionally, there is some latency associated with using LLM-based interfaces, as the models must process the data and query related information and tools. However, the models used in our evaluation provide answers in close to real-time with minimal latency, although this could increase under high concurrency if sufficient resources were not available. Clinical deployment of LLMs requires regulatory approval (Ong et al., 2024). Moreover, any AI system that informs clinical decisions must satisfy appropriate medical device regulations and data must be used appropriately, for example complying with GDPR in the EU. While we believe LLM-based systems are more robust than standalone LLMs, questions remain around data privacy and security. Such challenges could be addressed with open-source LLMs that run locally in secure compute environments managed by medical providers. While being able to update the sources of information is a key benefit of LLM-based interfaces, this introduces additional maintenance. Documents and tools could be automatically updated from a central resource, in cases where one exists. Failure to update systems as new guidelines and other resources are published may result in incorrect recommendations.

Finally, conducting user studies with clinicians is a critical next step in evaluating the effectiveness of LLM-based interfaces and an exciting direction for future work. While our quantitative assessment demonstrates the potential benefit of such systems, a limitation is the possibility of biases in the question sets. The questions used cover a broad range of topics and multiple use cases; however, they do not rigorously probe edge cases, such as poorly or ill-posed questions. The true value and limitations of LLM-based systems can only be fully understood through in-depth trials with the target users in real-world settings.

As AI in medicine continues to advance, further research into LLMs and their potential applications in healthcare could provide significant benefits. For example, LLMs could help alleviate the data burden that is contributing to clinician burnout, as well as streamline patient management processes. Furthermore, studies have demonstrated high usability of LLMs, even with limited experience (Skjuve et al., 2023), which is critical for successful clinical deployment. While we believe this paper represents an important first step, we are only scratching the surface of the potential of LLMs in healthcare. Ultimately, this line of work may significantly change the digital health landscape, enhancing the capabilities of clinicians and the quality of patient care.

## Data Availability

The datasets presented in this study can be found in online repositories. The names of the repository/repositories and accession number(s) can be found at: https://github.com/pauliusrauba/LLMs_interface.
