# Supplementary material for: Redefining digital health interfaces with large language models
Source: Front Artif Intell. 2025 Sep 26;8:1623339. doi: 10.3389/frai.2025.1623339 (PMC12511092; doi:10.3389/frai.2025.1623339)
Supplement: Supplementary file 1 [file Data_Sheet_1.pdf]

Redefining Digital Health Interfaces with Large Language Models

Fergus Imrie[1*], Paulius Rauba[2] and Mihaela van der Schaar[2*]

[1]Department of Statistics, University of Oxford, Oxford, United Kingdom.

[2]Department of Applied Mathematics and Theoretical Physics, University of Cambridge, Cambridge, United Kingdom.

*Corresponding author(s). E-mail(s): fergus.imrie@stats.ox.ac.uk; mv472@cam.ac.uk;
Contributing authors: pr501@cam.ac.uk;

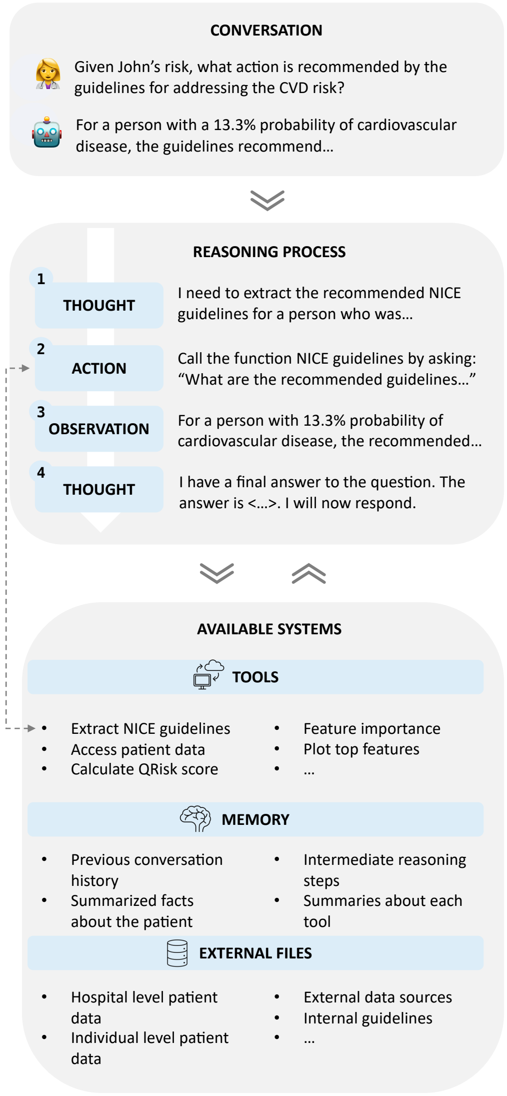

Fig. S.1: **Illustration of the process by which the LLM uses external tools.** The LLM is provided with the history of the interaction, including the current request. Using an iterative reasoning process, the LLM decides which, if any, tools are required and with what input ("Thought"). The LLM then uses the external tool ("Action") and receives the output ("Observation"). Finally, the LLM decides to answer the question ("Thought"), providing a response to the user.

Table S.1: **Performance of LLM-based interfaces using GPT-4o for CVD risk prediction.** Across a varied set of questions, our LLM-based system (Ours) significantly outperformed the same LLM used in a standalone manner (GPT-4o). For each representative question, we report the number of successes and questions, as well as the success rate.

	Representative questions	GPT-4o	Ours
Q1	Which features does the risk score use?	0/10 (0.0%)	10/10 (100%)
Q2	Why are these features included in the risk score?	20/21 (95.2%)	21/21 (100%)
Q3	How was the risk score validated?	3/10 (30.0%)	10/10 (100%)
Q4	What is the methodology underlying the risk score?	4/10 (40.0%)	10/10 (100%)
Q5	When do clinical guidelines recommend risk scoring?	2/10 (20.0%)	10/10 (100%)
Q6	What is the recommended risk score?	10/10 (100%)	10/10 (100%)
Q7	Who is the risk score suitable for?	10/10 (100%)	10/10 (100%)
Q8	What is the risk for this patient?	0/10 (0.0%)	10/10 (100%)
Q9	What characteristics led to the patient's risk?	0/10 (0.0%)	10/10 (100%)
Q10	What effect would changing this feature have on the risk?	1/10 (10.0%)	10/10 (100%)
Q11	What is recommended by the guidelines based on the risk?	14/16 (87.5%)	15/16 (100%)
	**Overall**	64/127 (50.4%)	126/127 (99.2%)

Table S.2: **Performance of LLM-based interfaces using GPT-4o for stroke risk prediction in atrial fibrillation patients.** Across a varied set of questions, our LLM-based system (Ours) significantly outperformed the same LLM used in a standalone manner (GPT-4o). For each representative question, we report the number of successes and questions, as well as the success rate.

	Representative questions	GPT-4o	Ours
Q1	Which features does the risk score use?	10/10 (100%)	10/10 (100%)
Q2	Why are these features included in the risk score?	12/14 (85.7%)	14/14 (100%)
Q3	How was the risk score validated?	3/10 (30.0%)	10/10 (100%)
Q4	What is the methodology underlying the risk score?	6/8 (75.0%)	7/8 (87.5%)
Q5	When do clinical guidelines recommend risk scoring?	1/10 (10.0%)	9/10 (90.0%)
Q6	What is the recommended risk score?	5/7 (71.4%)	7/7 (100%)
Q7	Who is the risk score suitable for?	7/7 (100%)	7/7 (100%)
Q8	What is the risk for this patient?	8/10 (80.0%)	10/10 (100%)
Q9	What characteristics led to the patient's risk?	9/10 (90.0%)	10/10 (100%)
Q10	What effect would changing this feature have on the risk?	9/10 (90.0%)	10/10 (100%)
Q11	What is recommended by the guidelines based on the risk?	9/10 (90.0%)	10/10 (100%)
	**Overall**	79/106 (74.5%)	104/106 (98.1%)

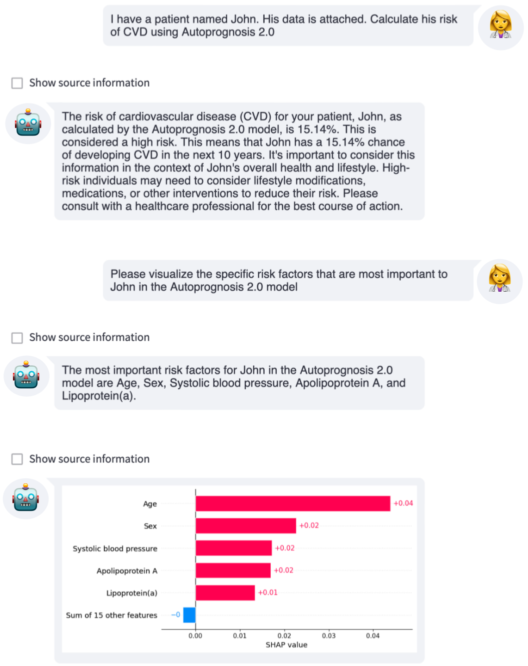

Fig. S.2: **Example interaction with an LLM-based interface.** After calculating the patient's risk using the AutoPrognosis model, the clinician can query why this prediction was issued using explainable AI.
